# Board informal hierarchy, strategic aggressiveness, and corporate ESG performance

**DOI:** 10.1371/journal.pone.0319264

**Published:** 2025-07-23

**Authors:** Jinye Xu, Yuanjie Xiao

**Affiliations:** School of Management of Shanghai University, Shanghai, China; The University of Texas at El Paso, UNITED STATES OF AMERICA

## Abstract

Based on the stakeholder theory, relationship contract theory, and other theories, this study examined how the board informal hierarchy influences corporate ESG (Environmental, Social, and Governance) performance, using Chinese A-share listed enterprises from 2010 to 2022 as the research samples. The results indicated that the clearer the board informal hierarchy, the better the corporate ESG performance. Furthermore, the board informal hierarchy can enhance the ESG performance by promoting more aggressive strategic initiatives. In situations characterized by high environmental uncertainty, heavy pollution industries, and state-owned enterprises, the positive impact of the board informal hierarchy on the corporate ESG performance is even stronger. This study provided a new perspective on the factors influencing the corporate ESG performance by examining the micro-level vertical structure within the board, enriching the content of ESG-related research.

## Introduction

Recently, green, low-carbon, and sustainable development have become an international consensus, drawing increased attention to issues such as environmental degradation and corporate misconduct. ESG (Environmental, Social, and Governance) performance has become central focus in discussions on global environmental initiatives. ESG not only involves ethics and social responsibility but also directly relates to the long-term sustainability and economic performance of companies. Under the guidance of China’s high-quality development strategy, ESG has become a key driver for enterprises to enhance competitiveness and achieve new milestones. In 2020, China set the dual carbon targets to achieve “carbon peak” by 2023 and “carbon neutrality” by 2060. The *White Paper on ESG Practices in China 2023* highlighted the need to continue practicing ESG principles and implementing sustainable development concepts, emphasize the acceleration of green transformation, deepen efforts in environmental pollution prevention, and enhance the ecosystem diversity, stability, and sustainability. From the perspective of enterprises, ESG performance enables managers to evaluate and compare their production and operational activities with peers. From the perspective of investors, ESG performance helps them better evaluate and select potential sustainable investment activities [1]. Enhancing ESG is conducive to exploring new market opportunities, promoting the growth of core businesses, and serving as an engine for innovation, market differentiation, and long-term value development [2].

ESG is closely related to global ecosystem protection and is regarded as an important tool for promoting high-quality sustainable economic and social development, as well as contributing to the goal of achieving shared prosperity. In terms of economic outcomes, ESG impacts a company’s ability to raise capital and attract investors, thereby enhancing company performance [3]. Strong ESG performance can reduce financial risks and increase corporate transparency [4]. In terms of influencing factors, Crace and Gehman [5] found that internal factors, such as the CEO and the company itself, were strong determinants of improving ESG performance. Companies involved in mergers and acquisitions can access more sustainable resources and are more likely to be scrutinized by governments and regulatory agencies, which positively influences their ESG performance [6]. Although there has been extensive research on ESG, studies focusing on the factors affecting ESG performance remain limited. China’s *Code of Corporate Governance for Listed Companies*, revised in 2018, stipulates that while improving corporate performance, the board of directors should also actively fulfill their social responsibilities, with an emphasis on green development and ecological civilization, thus promoting ESG practices. As the core of corporate governance, the board of directors plays a crucial role in promoting sustainable development and is a key entity in implementing ESG principles. Therefore, this study focuses on the board of directors as a central point of analysis to further explore the factors influencing ESG performance.

The board of directors plays a key role in supervising and advising management, as well as helping the company manage resources. Many scholars believe that to truly understand the internal governance of the board, more attention needs to be paid to the actual interactions within the board [7,8]. Due to the differing status and rights of board members, varying ranks often emerge among them, leading to the formation of an informal hierarchy. In China, where the culture of “authority obedience” prevails, individuals are highly sensitive to power and status, individuals are highly sensitive to power and status. As a result, hierarchical relationships within organizations significantly impact interpersonal interactions, which in turn play a critical role in shaping the overall operation and outcomes of the organization [9]. In China’s traditional “relationship-driven” society, board decisions are closely related to informal systems based on the board members’ statuses [10]. Jiang et al. [11] found that the clarity of the board’s informal hierarchy positively affects the relationship between changes in analysts’ recommendations and the extent of strategic changes. On the other hand, Jebran et al. [12] argued that the informal hierarchy structure among directors can exacerbate management’s tendency to conceal unfavorable information, thereby increasing the risk of future stock price crashes. The informal hierarchy within the board of directors can facilitate internal information flow, provide flexibility in responding to changes and challenges, and ensure that board decisions are more diverse and comprehensive [13]. This demonstrates that the informal hierarchy within the board plays a significant and indispensable role in corporate governance.

Currently, some scholars have examined the relationship between the board of directors and ESG performance. Garcia-Torea et al. [14] found a significant positive correlation between independent directors and corporate social responsibility committees with ESG disclosure, emphasizing the importance of corporate governance in promoting corporate social responsibility. Similarly, Birindelli et al. [15] empirically demonstrated that the larger the size of the board of directors, the better the ESG performance. However, these studies primarily focused on the horizontal structural characteristics of the board of directors, with limited research on how vertical hierarchies impact environmental, social responsibility, and corporate governance practices. Dong et al. [16] examined corporate green governance at a micro-level and found that both formal and informal hierarchies of the board of directors negatively impact the corporate green governance behavior. In more recent research, Vaughan and Koh [17] focused on the relational networks among board members and found that more closely connected boards could positively influence corporate social responsibility. While research on the impact of board structure has gradually grown, there is still no consensus on its effectiveness, making it necessary to further explore the impact of informal hierarchies formed by social capital imbalances and personal characteristics. Specifically, the impact and economic consequences of the informal hierarchies on corporate governance and decision-making require deeper investigation.

Based on the above analysis, academic research on the characteristics of boards of directors in relation to ESG remains insufficient, with inconsistent conclusions. Research on board hierarchy is not comprehensive, and there is ample room for exploring the influencing factors of ESG performance. Research on ESG development in China is still in its early stages. Studying the relationship between the hierarchical differences within corporate boards and corporate ESG performance is crucial for understanding how Chinese companies address the challenges of the ESG ecosystem during market-oriented transformation, and how they make decisions and formulate governance strategies within a relational society context. However, there is a lack of relevant research on this topic.

To address this gap, this paper utilized Stata software to collect and organize relevant data from Chinese listed companies from 2010 to 2022. It employed descriptive statistics, correlation analysis, regression analysis, robustness tests and further analyses of related research hypotheses. From the perspective of corporate governance effectiveness, the study found that the informal hierarchy of the board of directors has a positive impact on corporate ESG performance. Additionally, this paper explored the mediating role of strategic aggressiveness and the moderating effect of environmental uncertainty. The aim was to deepen the understanding of board structure and enrich the literature on factors affecting ESG performance, providing new insights for improving ESG performance of Chinese companies.

The contributions of this study were as follows. First, previous research has primarily focused on the impact of board composition, diversity, and independence on corporate governance. Johnson et al. [18] found that board composition is a key factor in influencing company performance, and board diversity has a positive impact on both internal and external outcomes [19]. Liu et al. [20] confirmed a positive correlation between board independence and company performance. This study, however, took a different perspective by examining the implicit order within the board, specifically the informal hierarchical structure. Through empirical analysis of its impact on corporate ESG performance, it deepened our understanding of the relationship between internal board mechanisms and corporate ESG performance, offering a novel perspective on the factors influencing corporate ESG performance and providing data-driven insights for advancing corporate sustainable development. Second, in a highly competitive industry environment, companies tend to adopt relatively aggressive strategies to respond to external pressures and gain competitive advantages [21]. Compared to conservative companies, those with aggressive strategies are more likely to implement environmental protection measures, assume greater social responsibility, and pursue long-term sustainable development to gain the recognition of relevant stakeholders and maximize corporate value [22,23]. However, no research has confirmed the positive role of strategic aggressiveness in the relationship between informal board hierarchy and ESG performance. Therefore, this study introduced strategic aggressiveness as a mediating variable to analyze how strategic decisions made by the informal board hierarchy further influence ESG performance.

## Theoretical analysis and research hypothesis

### Board informal hierarchy and ESG performance

According to stakeholder theory, enterprises are accountable to their stakeholders, and as the governing body of a company, the board of directors is responsible for respecting and safeguarding the legitimate rights and interests of all stakeholders [24]. As a result of board arrangements, the informal hierarchy within the board of directors plays a crucial role in corporate governance. The board informal hierarchy is a manifestation of informal systems that can have profound effects on the board and influence its operations and decisions, particularly in driving ESG practices [25]. The relational contract theory, based on mutual trust, respect, and cooperation, can promote effective communication and collaboration among employees within organizations, thereby enhancing organizational performance and employee satisfaction. This theory has important implications for organizational management, team collaboration, maintaining strong stakeholder relationships, contributing to the establishment of a positive work environment, and enabling organizations to better address internal and external challenges [26].

Firstly, based on the aforementioned relational contract theory, Johnson et al. [26] mentioned that informal hierarchies are more often established as a system based on trust and respect. The differential order formed within the board of directors can influence the relationships among board members, with those perceived to contribute more often enjoying higher levels of trust and respect [27]. They may easily influence board decisions as other members are more willing to collaborate with them and participate more actively in board affairs, facilitating informal communication and exchanges among members. ESG performance closely relates to a company’s competitive advantage in the market and long-term sustainability, often receiving significant attention from the board of directors [28]. Directors in higher positions tend to introduce ESG issues into board discussions, guiding board members to focus on environmental, governance, and related matters. Lower-ranking directors, driven by trust and respect, are more likely to actively reach a consensus, thereby improving decision-making efficiency and helping the company advance its ESG initiatives.

Secondly, senior board members often bring extensive strategic experience and resources due to their prestige and influence in the industry or political sphere, which can provide critical support for corporate decision-making. They typically have a broader industry network, enabling them to obtain critical information and establish strategic partnerships, thereby better understanding and addressing ESG challenges and taking related actions. On the other hand, junior directors are often willing to accept the opinions of senior directors, which can reduce internal friction and conflicts in decision-making, enhancing information integration and decision efficiency regarding ESG [29]. As ESG expands and deepens globally, the directors advocate for adopting more environmentally responsible practices, improving social responsibility initiatives, and strengthening governance standards to maintain and enhance their own reputation in the external environment. Particularly, senior directors, aiming to realize their own values and demonstrate forward-thinking capabilities, influence other board members to collectively focus on the company’s long-term sustainable development, thereby enhancing the company’s ESG performance.

Finally, the formal hierarchies within Chinese boards, tend to follow strict systems and procedures, this rigid structure may hinder innovation and flexibility, potentially leading to an over-reliance on formal processes and reducing board efficiency. In contrast, a clear informal hierarchy within the board can mitigate potential conflicts, foster team cohesion, and enhance internal organizational stability. These factors collectively influence internal collaboration, decision-making, and the advancement of ESG objectives. Compared to formal structures, informal hierarchies are better at minimizing opportunistic behavior among members and reducing governance issues [30]. As a complement to formal systems, informal structures can play a significant role in corporate governance [31]. Based on the above analysis, this paper proposed the following hypothesis:

**H1.** Board informal hierarchy has a positive impact on corporate ESG performance.

### Mediating role of strategic aggressiveness

Strategic aggressiveness refers to a company’s flexibility and adaptability to quickly adapt to external changes through offensive strategies, enabling it to identify opportunities and risks effectively. Furthermore, implementing aggressive strategies requires efficient execution capabilities to meet the company’s long-term objectives [32]. The board of directors is responsible for ensuring that the organization’s strategic choices align with its long-term goals and shareholder interests through effective cooperation and supervision. Additionally, they continuously adjust and optimize strategies in response to changes in the market and competitive environment to safeguard the company’s long-term development.

Therefore, the board of directors plays a crucial role in corporate strategy execution, managing risks, overseeing execution, and interacting with shareholders. According to organizational hierarchy theory, informal hierarchical structures can provide clear social order within the board of directors, creating favorable organizational and collaborative relationships. Members may exhibit higher levels of trust, information sharing, and problem-solving abilities, aiding in faster and higher-quality decision-making and reducing the likelihood of internal conflicts [33]. Additionally, senior directors leverage social resources and external relationships, including industry contacts, government relations, and investor relations, to provide crucial information about corporate development to the board through the exchange and sharing of internal information. They aim to help board members understand and support the purpose of corporate strategic decisions, thereby promoting discussions and adoption of aggressive strategies [34]. The presence of informal hierarchical structures facilitates smoother information flow among board members, reduces information blind spots, and ultimately enhances the formulation and execution of aggressive strategic decisions by the company [35].

Furthermore, capture a larger market share and expand into new areas of business, the board of directors often makes decisions that are more competitive and innovative. The decision-making process in this regard is partially influenced by informal hierarchies [36]. A clear informal hierarchy can create a conducive decision-making environment for the board of directors, providing clear guidance for strategic execution. Board members, aiming to realize their own values and adapt to the rapidly changing market and competitive pressures, often favor aggressive strategies [37]. Thus, this paper hypothesized that:

**H2a.** Board informal hierarchy is positively correlated with strategic aggressiveness.

Additionally, companies also need to adapt flexibly to the constantly changing external environment by timely adjusting strategies or reselecting strategies to maintain long-term success and competitive advantage [38]. On the one hand, considering the long-term sustainability of the enterprise and addressing ESG issues that stakeholders concern, adopting aggressive strategies makes it more likely for enterprises to incorporate ESG factors into their business plans and objectives by taking proactive actions in the ESG domain. According to resource dependency theory, organizations rely on external resources and environmental factors to support their operations. If the external environment demands higher ESG standards, such as stricter environmental regulations or social responsibility pressures, organizations may be influenced by these demands and need to make corresponding adjustments in their ESG performance. For companies adopting highly aggressive strategies, their ESG practices can enhance market competitiveness and increase opportunities and capabilities for resource acquisition. This not only contributes to the long-term sustainable development of the enterprise but also enhances the motivation to fulfill corporate social responsibility [39].

On the other hand, social pressure and public concern compel companies to actively manage their reputation. Under informal systems, it is easier to prompt companies to implement aggressive strategies, focusing on ESG factors to obtain better development opportunities [40]. This study argued that a well-defined power structure among directors facilitates effective resource exchange, communication, and discussion. As a result, directors can better understand both the internal and external environments of the company and are thus more likely to adopt proactive strategies that allow the company to respond effectively to external changes and maintain a long-term competitive advantage [41]. Aggressive strategic choices pursue innovation and explore new markets, which encourages companies to focus on improving ESG performance, thereby consolidating and enhancing their core competitiveness. Therefore, this paper hypothesized the following:

**H2b.** Strategic aggressiveness plays a mediating role between the board informal hierarchy and ESG performance.

### Moderating role of environmental uncertainty

According to resource dependence theory, enterprises need to acquire, manage, and rely on various resources to maintain their business activities. It emphasizes that changes and uncertainties in the external environment may have an impact on resource acquisition and utilization, requiring enterprises to constantly adjust their strategies. In highly uncertain environments, the board of directors is responsible for formulating and reviewing the company’s long-term development direction and strategic plans to ensure that the company can respond flexibly to changes [42]. Environmental uncertainty is usually characterized by complexity, randomness, and dynamics. When making corporate strategic decisions, the board of directors needs to pay close attention to environmental factors, consider how environmental uncertainty affects the company’s future development, and take appropriate measures in response [43].

In highly uncertain environments, information may become confused and inconsistent, making it difficult for the board of directors to identify and filter out truly valuable information, thereby reducing decision-making efficiency and effectiveness and increasing operational risks for the company. Under such circumstances, timely dissemination and effective sharing of information become crucial. Within the board informal hierarchy, members possess rich professional backgrounds and accumulate social resources from their external positions [44]. Integration and transmission of environmental changes from different sources and types, as along with ESG-related information, among members at different levels, facilitate a more comprehensive understanding of the external environment and the challenges faced by the company. This process facilitates the exchange of diverse opinions and suggestions, enhancing the decision-making effectiveness. High-level directors, based on their experience and insights, guide board members in identifying important issues, reducing unnecessary discussions and information overload, providing clear direction and advice on critical issues, and assisting board members in reaching consensus more quickly. The leadership and decision-making abilities of high-level directors can reduce delays and disputes in the decision-making process, strengthen coordination and cooperation, and facilitate swift responses to dynamic external environments in times of high environmental uncertainty.

In addition, in situations of environmental uncertainty, companies face increased social and environmental risks, necessitating flexible adjustments to their ESG strategies to adapt to changing environmental and societal demands. The informal hierarchy within the board influences the integration of ESG into corporate strategies and business decisions, thereby promoting long-term sustainability. This not only facilitates rapid development for companies in complex and dynamic markets but also contributes to the continual improvement of society and environmental sustainability. Therefore, this paper hypothesized as follows:

**H3.** Environmental uncertainty moderates the relationship between board informal hierarchy and ESG performance.

**Fig 1:**
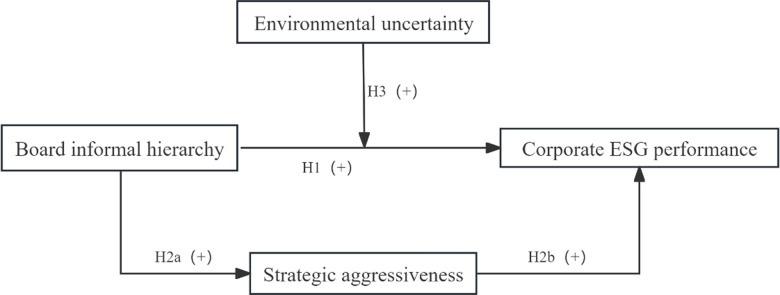
The mechanism model diagram.

Lastly, according to the above analysis, we summarized our study’s mechanism, as shown in Fig 1.

## Research design

### Sample

This study selected the listed companies in the Shanghai and Shenzhen A-shares in China, covering the period from 2010 to 2022, as the initial sample, which was processed as follows: (1) Excluding companies from the financial and insurance industries; (2) Excluding ST companies from the sample; (3) Excluding companies with missing values. As a result, this study obtained 20,404 annual observations from 3,716 companies. The sample data were obtained from the CSMAR database (China Stock Market & Accounting Research Database) and the Wind database. To eliminate outliers, all continuous variables are trimmed at 1% and 99% levels. Stata software (version 16) was used to conduct relevant data analysis.

### Variable measurements

#### Explained variables.

**ESG performance (ESG).** Drawing from the research methodology of Wang and Guo [45], the ESG rating data released by the Shanghai Sino-Securities Index Information Service Company (hereinafter referred to as the Huazheng Index) were used to measure the ESG performance of listed companies. The ratings range from AAA (the highest) to C (the lowest), with a total of nine levels. Each quarter was assigned a value from 9 to 1 in descending order, and the average of the scores from the four quarterly scores was taken as the proxy variable for the company’s ESG performance.

#### Explanatory variables.

**Board informal hierarchy (Gini).** The informal hierarchy within the board of directors refers to the structure of informal power and influence that emerges among board members. This hierarchy is typically formed due to differences in personal relationships, experience, reputation, seniority, expertise, and communication skills among board members [46]. This implicit status hierarchy reflects the varying levels of influence each board member holds within the organization. Some board members hold positions in multiple companies, gaining diverse operational and management experiences, which allows them to accumulate more valuable resources. As a result, their opinions and suggestions are often more authoritative, providing the company with distinct perspectives and strategic insights, thereby offering differentiated value to the board. Additionally, holding multiple directorships is commonly seen as a recognition and respect for board members’ capabilities and social influence, thereby increasing their status within the board [47]. Consequently, other board members are more inclined to consider the opinions and views of those with higher hierarchical status. This study adopted the methods of He and Huang [46] and Zhang [48] to measure board informal hierarchy using the Gini coefficient. The calculation formula for the Gini coefficient was as follows:


$$\text{Gini}=\frac{2cov\;（y，r_{y}）}{N\bar{y}}$$ (1)

where Gini represents the Gini coefficient; y denotes the number of outside directorships held by each director on the board; $r_{y}$represents the ranking of the number of directorships held; $\bar{y}$ is the average value of y; N represents the size of the board; cov(y,$r_{y}$) denotes the covariance between y and $r_{y}$; The Gini coefficient ranges from 0 to 1, with higher values indicating a clearer informal hierarchy within the board.

#### Mediating variables.

**Strategic aggressiveness (STR).** Following the methodologies outlined by Bentley et al. [40], this study adopted six dimensions to comprehensively measure strategic aggressiveness:1) The ratio of intangible assets to operating income (intangible assets were used as a substitute due to significant missing data on R&D expenses in the database); 2) The ratio of employee count to operating income; 3) The ratio of total sales and administrative expenses to operating income; 4) Operating income growth rate; 5) Employee volatility, calculated as the standard deviation of employee numbers over five years divided by the mean; 6) Capital intensity, calculated as the ratio of fixed assets to total assets.

The rolling five-year averages were calculated for all variables. Samples were grouped according to year and industry. The first five indicators were sorted from the largest to the smallest, while the sixth indicator was sorted from the smallest to the largest. The values were assigned scores of 4, 3, 2, 1, and 0, respectively, for each indicator. A total score was derived by summing the six indicators, creating a discrete variable ranging from 0 to 24, with higher the score indicating a stronger level of corporate strategic aggressiveness.

#### Moderating variables.

**Environmental uncertainty (EU).** To account for environmental uncertain, this study utilized Ordinary Least Squares (OLS) regression models to process sales revenue data from listed companies over the past five years. Abnormal sales revenue was estimated using the following formula:


$$Sale_{i,t}=\varphi_{0}+\varphi_{1}Year_{i,t}+\varepsilon_{i.t}$$ (2)

where Sale represents sales revenue, Year represents the year, and the residual ε represents abnormal sales revenue. Subsequently, abnormal sales revenue and its standard deviation for each company were estimated. The results obtained after removing industry influences can reflect environmental uncertainty.

#### Control variables.

This study incorporated control variables at both the board and company. At the board level, the control variables included board size (Board), independent director ratio (Indep), separation of the chairman and CEO roles (Dual), and majority shareholder ownership (Top1). At the company level, the control variables included return on assets (ROA), leverage ratio (Lev), firm size (Size), cash flow (Cashflow), firm growth (Growth), and company type (SOE). Specific variables were defined in Table 1.

**Table 1:** Definitions of variables.

Variable type	Variable Name	Symbols	Variable Definition
Explained variables	ESG performance	ESG	For the Huazheng ESG rating index, assign values from high to low as 9 to 1, then take the quarterly average.
Explanatory variables	Board Informal Hierarchy	Gini	$\text{Gini}=\frac{2cov\;（y，r_{y}）}{N\bar{y}}$
Mediating Variables	Strategic Aggressiveness	STR	Sum up the six indicators, as specified in the variable settings above.
Moderating Variabls	Environmental Uncertainty	EU	Environmental uncertainty adjusted for industry.
Control variables	Return on Assets	ROA	Net profit/total assets
	Leverage Ratio	Lev	It is measured by dividing long-term debt over total assets.
	Firm Size	Size	It is measured by taking the natural logarithm of total assets.
	Cash Flow Ratio	Cashflow	Net cash flow from operating activities/total assets
	Firm Growth	Growth	(Current year’s operating revenue—Previous year’s operating revenue)/Previous year’s operating revenue
	Board Size	Board	The natural logarithm of board size.
	Independent director ratio	Indep	Number of independent directors/ Total number of directors
	Separation of the Chairman and CEO Roles	Dual	If the CEO is also the chairman, the value is 1, otherwise it is 0
	majority shareholder ownership	Top1	Percentage of shareholding of the largest shareholder
	Company type	SOE	If it is a state-owned enterprise, the value is 1, otherwise it is 0.
	Year dummy	Year	Year dummy variables.
	Industry dummy	Industry	Industry code dummy variables.

### Models

Based on the variable measurements, the following theoretical models were constructed:

To test H1, this paper constructed Model (3) to examine the impact of informal board hierarchy on corporate ESG performance.


$$ESG_{i,t}=\alpha_{0}+\alpha_{1}Gini_{i,t}+\alpha_{2}Controls_{i,t}+Year_{i,t}+Ind_{i,t}+\varepsilon_{i,t}$$ (3)

To test H2a and H2b, this paper constructed Models (4) and (5) to examine the mediating effect of strategic aggressiveness on the relationship between informal board hierarchy and ESG performance.


$$Str_{i,t}=\beta_{0}+\beta_{1}Gini_{i,t}+\beta_{2}Controls_{i,t}+Year_{i,t}+Ind_{i,t}+\varepsilon_{i,t}$$ (4)

$$ESG_{i,t}=\gamma_{0}+\gamma_{1}Gini_{i,t}+\gamma_{2}Str_{i,t}+\gamma_{3}Controls_{i,t}+Year_{i,t}+Ind_{i,t}+\varepsilon_{i,t}$$ (5)

To test H3, this paper constructed Model (6) to examine the moderating effect of environmental uncertainty on the relationship between informal board hierarchy and ESG performance.


$$ESG_{i,t}=\varphi_{0}+\varphi_{1}Gini_{i,t}+\varphi_{2}EU_{i,t}+\varphi_{3}Gini_{i,t}\times EU_{i,t}+\varphi_{4}Controls_{i,t}+Year_{i,t}+Ind_{i,t}+\varepsilon_{i,t}$$ (6)

## Empirical results

### Descriptive statistics

The descriptive statistics results for both the dependent and independent variables were presented in Table 2. The maximum ESG score was 8, the minimum was 1, and the mean was 4.130. This indicated a significant variation in ESG scores among different sample companies, with most listed companies demonstrating good ESG performance. The maximum Gini value was 0.451 and the minimum was 0, indicating that while some boards of directors exhibit clear implicit hierarchical structures with a distinct distribution of influence among board members, there are also companies where board members maintain relatively equal standing. The mean of STR was 11.861, with a standard deviation of 4.487, indicating differences in the strategic aggressiveness choices among different companies, with the sample companies predominantly adopting an analytical strategic approach. The mean of EU was 3.035, with a standard deviation of 7.337, indicating significant uncertainty faced by the sample companies in the external environment.

**Table 2:** Descriptive statistics results.

Variables	N	Mean	Sd	Median	Min	Max
ESG	20,404	4.130	0.974	4.000	1.000	8.000
Gini	20,404	0.165	0.064	0.158	0.000	0.451
STR	20,404	11.861	4.487	12.000	0.000	24.000
EU	20,404	3.035	7.337	1.005	0.105	52.407
ROA	20,404	0.041	0.068	0.040	−0.398	0.254
Lev	20,404	0.416	0.200	0.408	0.027	0.925
Size	20,404	22.234	1.296	22.031	19.525	26.430
Cashflow	20,404	0.048	0.067	0.046	−0.224	0.257
Growth	20,404	0.178	0.395	0.119	−0.660	4.330
Board	20,404	2.125	0.197	2.197	1.609	2.708
Indep	20,404	0.377	0.054	0.364	0.273	0.600
Dual	20,404	0.297	0.457	0.000	0.000	1.000
Top1	20,404	0.340	0.148	0.319	0.081	0.758
SOE	20,404	0.316	0.465	0.000	0.000	1.000

### Correlation analysis

Table 3 showed the correlation coefficients of the main variables. From the table, it can be observed that the correlation coefficient between Gini and ESG was 0.031, which was significant at the 1% level, providing preliminary validation for hypothesis 1. The absolute values of the correlation coefficients among the main variables were all below 0.5, indicating that there was no severe multi-collinearity issue.

**Table 3:** Correlation matrix.

	ESG	Gini	STR	EU	ROA	Lev	Size
ESG	1						
Gini	0.031^***^	1					
STR	0.002	0.093^***^	1				
EU	0.118^***^	−0.090^***^	−0.160^***^	1			
ROA	0.235^***^	0.036^***^	0.066^***^	−0.009	1		
Lev	−0.085^***^	−0.110^***^	−0.219^***^	0.289^***^	−0.358^***^	1	
Size	0.188^***^	−0.107^***^	−0.237^***^	0.584^***^	0.002	0.513^***^	1
Cashflow	0.093^***^	0.012*	−0.038^***^	0.047^***^	0.401^***^	−0.158^***^	0.087^***^
Growth	0.005	0.018**	0.164^***^	0.033^***^	0.260^***^	0.010	0.026^***^
Board	0.029^***^	−0.355^***^	−0.119^***^	0.117^***^	0.0100	0.148^***^	0.266^***^
Indep	0.072^***^	0.159^***^	0.036^***^	0.074^***^	−0.013*	−0.003	0.010
Dual	−0.003	0.139^***^	0.147^***^	−0.070^***^	0.052^***^	−0.141^***^	−0.180^***^
Top1	0.107^***^	−0.055^***^	−0.144^***^	0.169^***^	0.133^***^	0.052^***^	0.195^***^
SOE	0.071^***^	−0.256^***^	−0.271^***^	0.227^***^	−0.084^***^	0.293^***^	0.373^***^
**(cont.)**	**Cashflow**	**Growth**	**Board**	**Indep**	**Dual**	**Top1**	**SOE**
Cashflow	1						
Growth	0.033^***^	1					
Board	0.030^***^	−0.015**	1				
Indep	0.002	−0.001	−0.546^***^	1			
Dual	−0.007	0.033^***^	−0.187^***^	0.108^***^	1		
Top1	0.098^***^	0.001	0.027^***^	0.047^***^	−0.048^***^	1	
SOE	−0.005	−0.071^***^	0.267^***^	−0.044^***^	−0.297^***^	0.246^***^	1

Note:

*** p < 0.01, **p < 0.05, * p < 0.1.

### Regression analysis

According to the model established in the previous section, multiple linear regression analysis was performed using Stata software. The main regression results of this study were shown in Table 4. The regression coefficients of Gini and ESG were both significantly positive at the 1% confidence level, confirming the positive influence of board informal hierarchy on the ESG performance. A clear informal power structure within the board helps reduce conflicts related to hierarchy, curb management’s self-interested behaviors, and provide clearer guidance on key company issues. Additionally, it introduces diverse perspectives and experiences, which helps in addressing ESG-related issues more effectively and promote the company’s ESG performance.

**Table 4:** Basic regression results.

Variables	(1)	(2)
	ESG	ESG
Gini	0.542^***^	0.768^***^
	(5.330)	(7.340)
ROA		2.809^***^
		(22.009)
Lev		−0.816^***^
		(−18.397)
Size		0.198^***^
		(28.677)
Cashflow		−0.364^***^
		(−3.313)
Growth		−0.101^***^
		(−5.680)
Board		0.192^***^
		(4.383)
Indep		1.543^***^
		(11.038)
Dual		0.032**
		(2.161)
Top1		0.213^***^
		(4.599)
SOE		0.098^***^
		(6.008)
_cons	3.511^***^	−1.636^***^
	(53.812)	(−9.286)
Year	YES	YES
Industry	YES	YES
Obs.	20,404	20,404
Adj_ $R^{2}$	0.035	0.156

Note:

*** p < 0.01, **p < 0.05, *p < 0.1. t-values are in parentheses.

Column (1) of Table 5 presented the test results for Model 2, showing a regression coefficient of 1.058 for strategic aggressiveness, which was significant at the 5% level. This finding indicated that board informal hierarchy can, to some extent, promote companies to adopt more aggressive strategies, supporting hypothesis H2a. Column (2) presented the results of the mediation effect test for Model 3, showing a positive and significant regression coefficient at the 1% level. This indicated that strategic aggressiveness partially mediates the relationship between board informal hierarchy and ESG performance, consistent with the theoretical hypothesis. This results suggested that, under the promotion of board informal hierarchy, companies tend to adopt aggressive strategies to adapt to market demands, increase innovation rates, and enhance core competitiveness. These strategies require appropriate resource integration and simultaneous attention to ESG performance. Column (3) presented the results of the moderating effect of environmental uncertainty, where the coefficient of the interaction term between board informal hierarchy and environmental uncertainty (Gini*EU) was 0.040, which was significant at the 5% level. This indicated that the higher the environmental uncertainty, the greater the influence of board informal hierarchy on corporate ESG performance, suggesting a positive moderating effect of environmental uncertainty on the relationship between the two variables and providing evidence in supporting hypothesis 3.

**Table 5:** Mediation and moderation effects.

Variables	(1)	(2)	(3)
	STR	ESG	ESG
Gini	1.058**	0.759^***^	0.669^***^
	(2.126)	(7.254)	(5.943)
STR		0.008^***^	
		(5.738)	
EU			0.006**
			(−2.154)
Gini*EU			0.040**
			(2.188)
ROA	−0.148	2.810^***^	2.808^***^
	(−0.261)	(22.094)	(21.950)
Lev	−3.336^***^	−0.788^***^	−0.816^***^
	(−16.934)	(−17.655)	(−18.360)
Size	−0.450^***^	0.202^***^	0.197^***^
	(−14.471)	(29.114)	(24.127)
Cashflow	−3.011^***^	−0.338^***^	−0.361^***^
	(−6.049)	(−3.083)	(−3.291)
Growth	1.881^***^	−0.117^***^	−0.101^***^
	(21.332)	(−6.518)	(−5.688)
Board	−0.152	0.193^***^	0.196^***^
	(−0.740)	(4.420)	(4.471)
Indep	1.925^***^	1.527^***^	1.554^***^
	(2.940)	(10.933)	(11.082)
Dual	0.584^***^	0.027*	0.032**
	(8.820)	(1.821)	(2.190)
Top1	−2.966^***^	0.238^***^	0.217^***^
	(−14.231)	(5.130)	(4.673)
SOE	−1.643^***^	0.112^***^	0.098^***^
	(−21.635)	(6.795)	(6.009)
_cons	24.185^***^	−1.840^***^	−1.624^***^
	(28.498)	(−10.235)	(−8.098)
Year	YES	YES	YES
Industry	YES	YES	YES
Obs.	20,404	20,404	20,404
Adj_ $R^{2}$	0.169	0.157	0.156

Note:

*** p < 0.01, **p < 0.05, *p < 0.1. t-values are in parentheses.

To validate the mediating role of strategic aggressiveness, this study referenced the research of Preacher et al. [49] and Wen [50], employing the Bootstrap sampling method for testing, with a sample size of 5,000. A bias-corrected confidence interval was used, and if the 95% confidence interval did not include zero, it would confirm the existence of a mediating effect between the independent and dependent variables. The results, as shown in Table 6, indicated that none of the confidence intervals included zero, thereby confirming that the mediating effects were significant. This further substantiates the mediating role of strategic aggressiveness in the relationship between informal board hierarchy and ESG performance.

**Table 6:** Analysis of Bootstrap mediation effect.

Effect	Observed Coef.	P value	95% Conf. Interval	Result
Indirect effect	0.125	0.004	[0.201, 0.324]	Significant
Direct effect	0.661	0.000	[0.455, 0.868]	Significant

Note: Number of bootstrap samples = 5,000.

### Robustness tests

#### Generation of lagged variables.

Given that the impact of board informal hierarchy on corporate ESG may not be immediately reflected in the current period but rather manifested in subsequent years, this study conducted a regression analysis using data with a one-period lag on board informal hierarchy to address endogeneity issues. The regression results were presented in Table 7. In column (1) of Table 7, the regression coefficient between board informal hierarchy and ESG performance was 0.5, which was significant at the 1% confidence level. This indicated that the board informal hierarchy contributes to improving corporate ESG performance through corporate governance practices. Columns (2)–(4) of Table 7 presented the regression results with the inclusion of moderating and mediating variables, respectively. The corresponding regression coefficients were positive and significant, further demonstrating the robustness of the results in this study.

**Table 7:** Regression results with informal board hierarchy lagged by one period.

Variables	(1)	(2)	(3)	(4)
	ESG	ESG	STR	ESG
L.Gini	0.500^***^	0.324**	0.668	0.498^***^
	(3.965)	(2.382)	(1.128)	(3.950)
EU		0.009^***^		
		(−2.941)		
Gini*EU		0.069^***^		
		(3.181)		
STR				0.004**
				(2.501)
ROA	3.877^***^	3.875^***^	3.066^***^	3.871^***^
	(24.334)	(24.280)	(4.121)	(24.332)
Lev	−0.781^***^	−0.780^***^	−3.577^***^	−0.765^***^
	(−14.884)	(−14.837)	(−14.998)	(−14.476)
Size	0.206^***^	0.204^***^	−0.577^***^	0.208^***^
	(25.311)	(21.193)	(−15.642)	(25.392)
Cashflow	−0.142	−0.137	−5.060^***^	−0.123
	(−1.087)	(−1.054)	(−8.435)	(−0.941)
Growth	−0.031	−0.032	1.955^***^	−0.039*
	(−1.528)	(−1.569)	(17.780)	(−1.910)
Board	0.106**	0.112**	0.019	0.106**
	(2.061)	(2.192)	(0.076)	(2.061)
Indep	1.552^***^	1.563^***^	2.889^***^	1.540^***^
	(9.631)	(9.672)	(3.711)	(9.555)
Dual	0.038**	0.038**	0.578^***^	0.036**
	(2.172)	(2.184)	(7.286)	(2.042)
Top1	0.062	0.068	−2.770^***^	0.075
	(1.145)	(1.256)	(−11.303)	(1.379)
SOE	0.195^***^	0.194^***^	−1.630^***^	0.202^***^
	(10.253)	(10.218)	(−18.362)	(10.533)
_cons	−1.662^***^	−1.602^***^	26.079^***^	−1.764^***^
	(−8.033)	(−6.795)	(25.918)	(−8.353)
Year	YES	YES	YES	YES
Industry	YES	YES	YES	YES
Obs.	14,805	14,805	14,805	14,805
Adj_ $R^{2}$	0.180	0.181	0.197	0.181

Note:

*** p < 0.01, **p < 0.05, *p < 0.1. t-values are in parentheses.

#### Propensity score matching (PSM).

To avoid endogeneity issues caused by sample self-selection, this study also employed propensity score matching (PSM) for robustness testing. The board informal hierarchy was divided by industry and year and grouped based on median values. Those above the median constituted the treatment group, while those below the median formed the control group. In accordance with established research practices, the control variables in this study were used as covariates for 1:1 nearest-neighbor matching. A regression analysis was then conducted on the matched samples. As shown in Table 8, the regression coefficient of the board informal hierarchy with ESG was 0.789, which was significant at the 1% level. The results for moderation and mediation effects remain consistent with earlier validation, thereby reaffirming this study’s findings.

**Table 8:** Test results of propensity score matching.

Variables	(1)	(2)	(3)	(4)
	ESG	ESG	STR	ESG
Gini	0.789^***^	0.635^***^	1.561**	0.776^***^
	(5.419)	(3.996)	(2.279)	(5.325)
EU		−0.009**		
		(−2.155)		
Gini*EU		0.062**		
		(2.243)		
STR				0.008^***^
				(4.063)
ROA	2.971^***^	2.968^***^	0.005	2.971^***^
	(16.560)	(16.516)	(0.006)	(16.589)
Lev	−0.800^***^	−0.800^***^	−3.083^***^	−0.774^***^
	(−12.566)	(−12.539)	(−11.068)	(−12.101)
Size	0.201^***^	0.200^***^	−0.482^***^	0.205^***^
	(20.203)	(17.238)	(−10.947)	(20.530)
Cashflow	−0.329**	−0.324**	−3.057^***^	−0.303*
	(−2.095)	(−2.062)	(−4.376)	(−1.932)
Growth	−0.106^***^	−0.107^***^	1.947^***^	−0.123^***^
	(−4.235)	(−4.270)	(14.699)	(−4.845)
Board	0.299^***^	0.301^***^	−0.243	0.301^***^
	(4.235)	(4.262)	(−0.766)	(4.271)
Indep	1.821^***^	1.816^***^	2.473**	1.800^***^
	(8.824)	(8.777)	(2.546)	(8.728)
Dual	0.046**	0.046**	0.575^***^	0.041**
	(2.210)	(2.237)	(6.184)	(1.970)
Top1	0.338^***^	0.342^***^	−2.487^***^	0.359^***^
	(5.076)	(5.129)	(−8.487)	(5.396)
SOE	0.114^***^	0.113^***^	−1.706^***^	0.128^***^
	(5.034)	(5.029)	(−16.246)	(5.611)
_cons	−2.101^***^	−2.049^***^	24.549^***^	−2.308^***^
	(−7.928)	(−6.904)	(19.741)	(−8.563)
Year	YES	YES	YES	YES
Industry	YES	YES	YES	YES
Obs.	10,384	10,384	10,384	10,384
Adj_ $R^{2}$	0.167	0.167	0.166	0.168

Note:

*** p < 0.01, **p < 0.05, *p < 0.1. t-values are in parentheses.

#### Instrumental variable (IV).

To further address the impact of endogeneity, the instrumental variable (IV) method was used and the results were re-examined using the two-stage least squares method (2SLS). Following the instrumental variable construction approaches of Shakil [51], Zhu et al. [52] and Hoang et al. [53], the average informal hierarchy of directors in other listed companies in the same industry and year was selected as the instrumental variable (Gini_mean). Since the informal hierarchy of a single company’s board may be influenced by other companies in the same industry and year, but the informal hierarchy of other companies’ boards is unlikely to affect an individual company’s ESG performance, this choice satisfied the theoretical requirements for both exogeneity and relevance of the instrumental variable. The instrumental variable also passed the weak instrument and under-identification tests. Column (1) of Table 9 presented the first-stage regression results, where the Gini_mean coefficient was significantly positive, indicating a strong correlation between the instrumental variable and the endogenous variable. Column (2) of Table 9 presented the second-stage regression results, demonstrating a significantly positive correlation between Gini and ESG at the 1% significance level, confirming the robustness of the findings after accounting for the endogeneity issue.

**Table 9:** Test results of instrumental variable.

Variables	(1)	(2)
	Gini	ESG
Gini_mean	0.013^***^	
	(13.107)	
Gini		0.605^***^
		(7.340)
ROA	0.002**	2.712^***^
	(2.138)	(21.103)
Lev	0.000	−0.817^***^
	(0.172)	(−18.435)
Size	−0.000^***^	0.203^***^
	(−2.993)	(29.322)
Cashflow	0.001^***^	−0.428^***^
	(1.076)	(−3.885)
Growth	0.000	−0.106^***^
	(0.849)	(−5.967)
Board	0.000	0.174^***^
	(1.341)	(4.024)
Indep	−0.001**	1.586^***^
	(−1.282)	(11.317)
Dual	−0.000	0.033**
	(−0.203)	(2.240)
Top1	0.000	0.206^***^
	(0.338)	(4.437)
SOE	0.000*	0.087^***^
	(1.811)	(5.406)
_cons	0.011^***^	−2.309^***^
	(10.193)	(−10.613)
Year	YES	YES
Industry	YES	YES
Obs.	20,404	20,404
Adj_R^2^	0.526	0.156

Note:

*** p < 0.01, **p < 0.05, *p < 0.1. t-values are in parentheses.

#### Additional analysis.

Currently, China has not yet implemented unified and mandatory standards for ESG disclosure. However, mandatory disclosure requirements for ESG information have been proposed for enterprises with high levels of pollution. Therefore, compared to non-heavily polluting enterprises, heavily polluting enterprises, due to their greater environmental impact and higher public scrutiny, are often subject to stricter environmental regulations and harsher penalties. They are required to bear more social responsibility, which compels them to focus more on sustainable development, voluntarily adhere to various ESG reporting standards, and improve their ESG performance.

Therefore, this paper conducted a group analysis of industries based on whether an industry is classified as heavily polluting. Columns (1) and (2) of Table 10 showed the regression results. In the heavily polluting industry group, the regression coefficient of Gini was 1.338, which was significant at the 1% level. In the non-heavily polluting industry group, the regression coefficient of Gini was 0.551, which was also significant at the 1% level. These findings suggest that in heavily polluting industries, the board informal hierarchy within the board of directors plays greater emphasis on corporate green environmental governance, strengthen supervision and management, and foster a sustainable and high-quality ESG development situation for the enterprise.

**Table 10:** Results of heterogeneity analysis.

Variables	(1)	(2)	(3)	(4)
	ESG	ESG	ESG	ESG
	Heavily polluting	non-heavily polluting	State-owned	Non-state-owned
Gini	1.338^***^	0.551^***^	1.410^***^	0.614^***^
	(5.591)	(4.723)	(6.804)	(5.036)
ROA	3.034^***^	2.765^***^	2.351^***^	2.879^***^
	(8.511)	(19.914)	(8.298)	(19.962)
Lev	−0.913^***^	−0.801^***^	−0.738^***^	−0.816^***^
	(−9.073)	(−16.122)	(−9.552)	(−14.660)
Size	0.171^***^	0.211^***^	0.246^***^	0.176^***^
	(11.363)	(26.777)	(22.053)	(18.939)
Cashflow	−0.752^***^	−0.175	−0.451**	−0.302**
	(−3.004)	(−1.429)	(−2.250)	(−2.315)
Growth	−0.106**	−0.105^***^	−0.120^***^	−0.091^***^
	(−2.566)	(−5.229)	(−3.333)	(−4.506)
Board	0.433^***^	0.113**	0.116*	0.209^***^
	(4.572)	(2.301)	(1.682)	(3.638)
Indep	1.292^***^	1.642^***^	1.641^***^	1.380^***^
	(4.151)	(10.494)	(7.442)	(7.532)
Dual	0.065*	0.025	−0.071*	0.042^***^
	(1.836)	(1.547)	(−1.908)	(2.581)
Top1	0.267^***^	0.218^***^	0.044	0.214^***^
	(2.639)	(4.169)	(0.548)	(3.687)
SOE	0.032	0.120^***^		
	(0.912)	(6.465)		
_cons	−1.253^***^	−1.811^***^	−2.726^***^	−0.926^***^
	(−3.348)	(−9.065)	(−10.618)	(−3.672)
Year	YES	YES	YES	YES
Industry	YES	YES	YES	YES
Obs.	4,786	15,502	6,450	13,954
Adj_ $R^{2}$	0.122	0.172	0.214	0.136

Note:

*** p < 0.01, **p < 0.05, *p < 0.1. t-values are in parentheses.

State-owned enterprises are the core driving force behind the national economic development. Compared to non-state-owned enterprises, they typically enjoy a more stable positions and competitive advantages within the national economic system, enabling them to access more government resources and policy support. They play a crucial role in economic activities and contribute to economic growth. This paper explored the influence of board informal hierarchy on the ESG performance across different ownership structures of enterprises.

The regression results in columns (3) and (4) of Table 10 showed that in the state-owned enterprise group, the regression coefficient of Gini was 1.41, while in the non-state-owned enterprise group, it was 0.614, both significant at the 1% level. These results indicated that state-owned enterprises have certain political affiliations, which can enhance the social characteristics of board members and strengthen the level of board informal hierarchy. This allows enterprises to have better access to social capital and resources, support ESG-related activities, and create more momentum and opportunities, thus promoting the long-term sustainable development of the enterprises.

## Conclusion

As the primary body of corporate governance, the board of directors plays a crucial role in ensuring corporate responsibility and business ethics, shaping corporate culture, overseeing the achievement of strategic objectives, and influencing strategic decision-making within the companies. This article aimed to explore the relationship between the board informal hierarchy and ESG factors, as well as the mechanisms underlying the relationship.

The conclusions were as follows: First, there is a significantly positive correlation between the board informal hierarchy and corporate ESG performance. Second, the board informal hierarchy can promote the development of ESG by increasing the strategic aggressiveness of the company. Third, environmental uncertainty has a significantly positive moderating effect on the relationship between the board informal hierarchy and ESG performance. Fourth, the impact of the board informal hierarchy on ESG performance is more pronounced in heavily polluting industries and state-owned enterprises. These conclusions provide evidence and insightful recommendations for the board of directors to enhance ESG performance, assisting companies in building robust governance systems and improve corporate governance efficiency. Furthermore, advancing ESG initiatives enables companies to better fulfill their social responsibilities, strengthen their social reputation, and promot sustainable development.

## Discussion

Many studies have investigated horizontal board structures such as board size and independence. Cucari et al. [54] asserted that the average level of the board and the diversity of independent directors have an important impact on ESG disclosure. Boubaker et al. [55] also found that board characteristics had a positive impact on ESG performance. This paper adopted a board governance perspective, focusing on the differences in social resources, knowledge, experience, and the interactive behaviors among board members. It explored the implicit hierarchy within the board, and deepened the understanding of the relationship between the internal mechanism of the board and the ESG performance of enterprises. At the same time, it expanded on the economic consequences of board governance, helping companies better understand how to improve corporate ESG performance by considering both formal and informal structures of the board.

Research on the informal level of the board of directors has primarily emphasized regulatory mechanisms, with limited attention to its mediating role within this dynamic. Liu et al. [56] employed the independence of the supervisory board as a moderating variable and found that it can reduce the risk of future stock price declines caused by a high degree of board informality. Building upon the investigation of the relationship between board informal hierarchy and corporate ESG performance, this study introduced strategic aggressiveness as a mediating variable. It established a path model “board informal hierarchy - strategic aggressiveness - corporate ESG performance” to examine the interaction among the three factors. Drawing from organizational hierarchy theory, resource dependence theory, and other frameworks, this model provided a more comprehensive understanding of how board informal hierarchy influences corporate ESG performance.

Examining the relationship between differences in board informal hierarchy and corporate ESG performance is crucial for understanding how Chinese companies address the challenges of the ESG ecosystem during marketization transitions. It also offers valuable insights into how governance strategies are formulated within a relational society context. Furthermore, this study provides a new perspective for investigating the factors influencing corporate ESG performance and offers practical insights to drive companies towards sustainable development.

However, this study has several limitations. First, within board interactions, senior directors may exhibit biases and self-interest motivations when exercising their power. Due to data constraints, this issue cannot be fully addressed, indicating a need for further investigation. Second, this study focuses solely on the informal hierarchical structure, overlooking the combined of formal and informal hierarchies on ESG performance. Since formal and informal structures coexist within the board of directors, future research could explore their alternative and complementary roles. Third, despite conducting a series of robustness tests during the research process, it is impossible to completely eliminate endogeneity issues. Further research is required to address these potential issues.
